# Risk Factors and Changes of Peripheral NK and T Cells in Pulmonary Interstitial Fibrosis of Patients with Rheumatoid Arthritis

**DOI:** 10.1155/2019/7262065

**Published:** 2019-12-01

**Authors:** Na-Lin Lai, Wen Jia, Xia Wang, Jing Luo, Guang-Ying Liu, Chong Gao, Xiao-Feng Li, Jian-Fang Xie

**Affiliations:** ^1^Department of Rheumatology, The Second Hospital of Shanxi Medical University, 382 Wuyi Road, Taiyuan, Shanxi 030001, China; ^2^Department of Pathology, Brigham and Women's Hospital, Harvard Medical School, Boston, USA

## Abstract

**Objective:**

The absolute and relative changes of peripheral NK and T subsets are unclear in rheumatoid arthritis (RA) associated with pulmonary interstitial fibrosis (RA-ILD). To investigate the clinical risk factors, especially the changes of lymphocyte subsets, in RA-ILD in order to make early diagnosis and achieve prevention of the pulmonary interstitial lesions.

**Methods:**

A total of 100 RA and 100 RA-ILD patients were enrolled. Rheumatoid factor, anti-cyclic citrulline peptide antibody, erythrocyte sedimentation rate, immunoglobulin, and C-reactive protein were examined. The percentage and absolute number of NK, T, B, Treg, Th1, Th2, and Th17 cells in peripheral blood were determined by flow cytometry.

**Results:**

RA-ILD is more common in older and male RA patients and/or those with higher autoantibody titers. Flow cytometry showed that the absolute and relative numbers of CD56+ NK cells were significantly higher in RA-ILD (280.40 ± 180.51 cells/*μ*l vs. 207.66 ± 148.57 cells/*μ*l; 16.62 ± 8.56% vs. 12.11 ± 6.47%), whereas the proportion of T cells and CD4+ T cells was lower in peripheral blood of RA-ILD patients (69.82 ± 9.30%; 39.44 ± 9.87 cells/*μ*l) than that in RA patients (74.45 ± 8.72%; 43.29 ± 9.10 cells/*μ*l).

**Conclusions:**

The occurrence of RA-ILD is closely related to the older male patients with high titer of various self-antibodies. Imbalance of CD3−CD56+ NK cells and T cells with other subsets were found in RA-ILD patients, which, together with older age, male, and high levels of autoantibodies should be considered as risk factors of pulmonary interstitial lesions.

## 1. Introduction

Rheumatoid arthritis (RA) is a common autoimmune disease characterized by chronic synovitis. Persistent or recurrent episodes of synovitis can lead to joint bone destruction, dysfunction, and even disability. However, RA is not limited to joint tissue and multiple organs and tissues can also be involved. Among them, the lung that has abundant connective tissue and blood supply is one of the major organs affected by RA.

Interstitial lung disease (ILD) is a term for a group of progressive fibrous lesions involving pulmonary parenchyma [1, 2]. However, the presence of ILD has been largely ignored in the management of RA, mainly because related symptoms such as cough and dyspnea are subclinical and unspecific in most patients [1]. Because of the lack of specific disease indicators and obvious early clinical presentations, the diagnosis and treatment is not timely, resulting in low quality of life and high mortality in RA-ILD patients. Some studies show that the average survival time of patients with RA-ILD is less than 3 years [3].

Although the pathogenesis of RA-ILD remains poorly defined, early identification and institution of antifibrotic therapy in other models of fibrosing disorders has actually led to amelioration of disease progression [4]. Given that early recognition and treatment of RA-ILD is of paramount importance to potentially slow/alter the disease course, the discovery and validation of risk factors that can enhance our ability to diagnose early stage RA-ILD and/or predict response to treatment in clinical trials have garnered significant attention. Previous studies have shown that NK cells play a key role in the pathogenesis of autoimmune liver disease [5] and acute lung injury [6]. It can induce antifibrosis signal in the liver and lungs. Some studies have shown that above T lymphocytes may be involved in the pathogenesis of interstitial pneumonia [7, 8]. The absolute and relative changes of peripheral NK and T subsets are unclear in patients with RA-ILD.

Based on the analysis of the clinical factors corelated to RA-ILD, the aim of this study explored clinical presentations in early disease, which may be a precursor to pulmonary fibrosis for clinical diagnosis and treatment of RA-ILD.

## 2. Materials and Methods

### 2.1. Patient Data

Patients with a confirmed diagnosis of RA were selected from the clinical database of the Department of Rheumatology, Second Hospital of Shanxi Medical University, from June 2017 to April 2019. All the patients met the RA classification standard of the American Society of Rheumatology (ACR) in 1987 [4], excluding those with the following diseases: (1) patients with chronic respiratory diseases such as pulmonary heart disease, chronic obstructive pulmonary disease, bronchial asthma, bronchiectasis, chronic obstructive pulmonary disease (COPD), bronchial asthma, bronchiectasis, and so on; (2) those with tuberculosis or previously infected with tuberculosis; (3) those with pulmonary nodules or tumors; (4) those with other heart, kidney, liver, or other organ dysfunction; and (5) those with heavy smoking and drinking for a long time. The HRCT findings of RA-ILD patients were interstitial pneumonia and fibrosis with or without pulmonary dysfunction. The age, gender, disease duration, and treatment of patients were recorded. Erythrocyte sedimentation rate (ESR) was measured by the Westergren method, and rheumatoid factor (RF), antibodies to cyclic citrullinated peptides (ACPA), C-reactive protein (CRP), and immunoglobulin IgG, IgA, and IgM were determined by immune turbidimetry. Our study was approved by the Medical Ethics Committee of Shanxi Medical University (2018LL328).

### 2.2. Detection of Peripheral Lymphocytes and CD4 T-Cell Subsets by Flow Cytometry

#### 2.2.1. Reagent

Stimulin, ionomycin, golgi blocker, fetal bovine serum, and RPMI 1640 medium were all purchased from SIGMA Company. Absolute count microspheres-Trucount™ tube, hemolysin, Multitest CD3−FITC/CD8−PE/CD45−PercP/CD4−APC kit, Multitest CD3−FITC/CD16+56−PE/CD45−PercP/CD19−APC kit, and monoclonal antibody CD4−FITC, IL-4-PE, IFN-*γ*-APC, IL-17-PE, CD25−APC, and FOXP3−PE were selected from BD, USA.

#### 2.2.2. Detection of Peripheral Lymphocyte Subsets

In brief, after adding 50 *μ*l of fully mixed anticoagulated blood to the Trucount tubes A and B, 20 *μ*l CD3FITC/CD8PE/CD45PercP/CD4APC antibody and 20 *μ*l CD3FITC/CD16+56−PE/CD45PercP/CD19APC antibody were added to the tubes A and B, respectively, followed by vortexing, and the tubes were kept at room temperature for 15 minutes. Later, 450 *μ*l XFACS hemolysin was added and was mixed well and kept at room temperature for 15 minutes for flow cytometric analysis. Finally, MultiSET software was used to obtain 15000 cells for testing.

#### 2.2.3. Detection of CD4 T-Cell Subsets

To prepare cells for flow cytometry, 80 *μ*l of anticoagulant blood was cultured in 10 *μ*l PMA (final concentration 30 ng/ml), 10 *μ*l ionomycin (final concentration 750 ng/ml), and 1 *μ*l GolgiStop for 5 hours in a CO_2_ incubator at 37°C. Then, the cells were divided into two tubes. The anti-human CD4−FITC was added to both tubes A and B. After incubation for 30 minutes at room temperature, the tubes were mixed with freshly prepared fixation/permeabilization solution and vortexed and incubated at 4°C for 30 minutes in the dark. IL-4-PE and IFN-*γ*-APC were added to tube A and anti-human IL-17-PE to tube B and were incubated at room temperature for 30 minutes in the dark, washed with PBS, and analyzed by flow cytometry. Another anticoagulant blood (80 *μ*l) was incubated with CD4−FITC and CD25−APC at room temperature for 30 minutes in the dark. Then, the cells were treated with 1 ml freshly prepared fixation/permeabilization solution at 4°C for 30 minutes in the dark and then stained with anti-human FOXP3 antibody at room temperature for 30 minutes in the dark, washed with PBS, and detected by a flow cytometer (Calibur, BD, USA) within 24 hours. The relative percentage was analyzed by CellQuest software. The formula for calculating the absolute number of cells in each subgroup is absolute count of cells = percentage of positive cells in each subgroup × CD4 T-cell absolute number (cells/*μ*l).

### 2.3. Statistical Analysis

The data were analyzed by SPSS19.0 statistical software. Comparisons were performed by paired and unpaired Student's *t*-test if the values were normal and by Wilcoxon rank-sum test or Mann–Whitney test if the values did not follow a normal distribution. Results were expressed as mean ± standard deviation ($\bar{x}\pm s$). The count data were expressed as a count, and the *χ*^2^ test was used for comparison between the groups. Factors with significant difference were further tested in the binary logistic regression model to adjust confounders. The difference was statistically significant with *P* < 0.05.

## 3. Result

### 3.1. General Features of RA Patients with ILD

**Figure 1:**
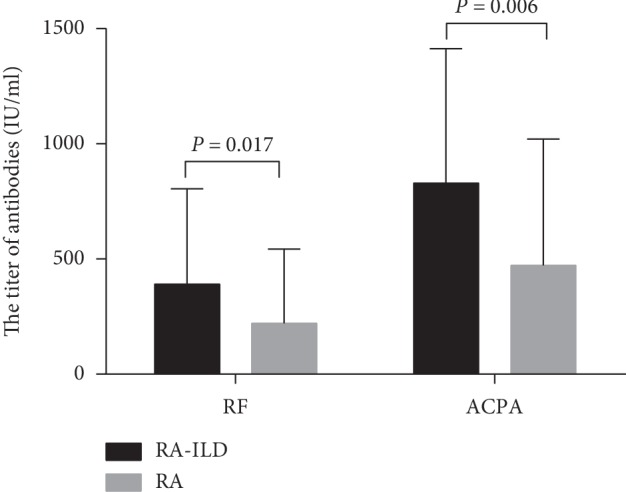
Comparison of autoantibody titers between RA-ILD and RA patients. The titers of RF and ACPA in the RA-ILD group are significantly higher than those in the RA group. The data are shown as the means, and the error bars represent the SD. Shown are the significant differences assessed by the binary logistic regression model.

356 cases were excluded, and 200 patients were eventually included. The total of 200 patients were further classified into the RA (*n* = 100) and RA-ILD (*n* = 100) group. In our retrospective study of RA-ILD patients, the proportion of male patients was 43%. Although most of the RA patients were female, the proportion of males in the RA-ILD group was higher than that in the RA group (*P*=0.011). The average age of RA-ILD patients (63.89 ± 8.99) was older than that of the RA group (53.26 ± 13.24) (*P* < 0.0001). Patients in both groups have been on medical therapy, including nonsteroidal anti-inflammatory drugs, glucocorticoids, disease-modifying antirheumatic drugs, and/or biological agents, but there were no significant differences. RF in the RA-ILD group was significantly higher than that in the RA group (391.05 ± 413.34 IU/ml vs. 221.02 ± 321.53 IU/ml, *P* < 0.0001). ACPA in the RA group is lower than that in the RA-ILD group (471.57 ± 549.60 IU/ml vs. 828.77 ± 584.23, *P* < 0.0001) (Figure 1). There was no significant difference in inflammatory markers (ESR and CRP) and immunoglobulins IgG, IgA, and IgM between the two groups (*P* > 0.05) (Table 1).

### 3.2. Comparison of Peripheral Lymphocyte Subsets in RA Group and RA-ILD Group

**Figure 2:**
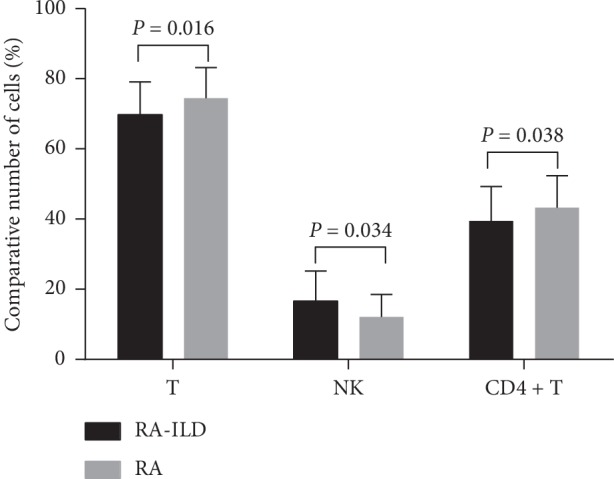
Relative counts of CD3−CD56+ NK, T cells, and CD4+ T cells between RA-ILD and RA patients. Comparing the peripheral lymphocyte subsets between RA-ILD and RA patients, the percentage of CD3−CD56+ NK cells in the RA-ILD group is higher than that in the RA group. The number of T cells and CD4+ T cells in RA-ILD group is lower than that in the RA group. The data are shown as the means, and the error bars represent the SD. Shown are the significant differences assessed by the binary logistic regression model.

In comparing the peripheral lymphocyte subsets between RA-ILD and RA patients, the percentage and absolute number of CD3−CD56+ NK cells in the RA-ILD group (16.62 ± 8.56%; 280.40 ± 180.51 cells/*μ*l) were higher than those in the RA group (12.11 ± 6.47%; 207.66 ± 148.57 cells/*μ*l) (*P* < 0.0001; *P*=0.002). Total T cells (69.82 ± 9.30%) were lower than those of the RA patients (74.45 ± 8.72%) (*P* < 0.0001). The number of CD4+ T cells in the RA-ILD group (39.44 ± 9.87%) was lower than that in the RA group (43.29 ± 9.10%) (*P*=0.005). However, the levels of B, CD8+ T, Th1, Th2, Treg, and Th17 cells were not significantly lower in the RA-ILD group than in the RA group (Tables 2 and 3 and Figure 2).

### 3.3. Multivariate Analyses of Factors Associated with RA-ILD

To exclude the effects of mismatched gender and age, a regression analysis was performed. The results showed that RF, ACPA, NK cells, T cells, and CD4+ T cells were the risk factors associated with RA-ILD (Table 4).

## 4. Discussion

Although the cognition of RA-ILD has become more and more profound and comprehensive, the pathogenesis is still unclear. RA itself is a risk factor for pulmonary interstitial fibrosis, but only about 10% of RA patients have pulmonary interstitial disease during the disease [9]. It is suggested that the pathogenesis of RA-ILD may be related to many other factors, such as environment, serology, gene, and so on. The demographic factors of the development of RA to RA-ILD include older age, male, and long disease duration [3, 9], which may be related to the higher number of men smoking because smoking is an established risk factor for the development of RA as well as the development of RA-ILD [10, 11]. In our study, the average age of the RA-ILD group was also higher than that of the RA group, which was consistent with the results of the previous study as RA-ILD was associated with advanced age. However, there was no significant difference in the mean disease duration between the two groups, which may due to the insufficient number of our patients.

We found that the titers of RF and ACPA in RA-ILD patients were significantly higher than those in the RA group. The pathological process of RA is complicated, but the high level of autoantibody in blood is closely related to severe clinical manifestations and joint damage and even increases the mortality rate of the body [12, 13], which is mainly related to RF, ACPA, and their immune complexes with self-proteins [14, 15]. The immune complex formed by ACPA and RF interacts with self-antigens, which can enhance inflammatory response and destructive response [13]. Moreover, the citrullinated peptide chain was found in pulmonary tissue and synovial fluid biopsy [16]. A recent study, which compared anti-citrulline-heat shock protein (HSP90) antibodies in lung lavage fluid and serum in patients with RA-ILD, found that pulmonary microenvironment plays a key role in the formation of ACPA and further induces an immune response in the lungs [17].

Some investigators found that smoking increases the likelihood of producing ACPA at the onset of RA, and there is an increased prevalence of lung disease (predominately ILD) in patients with high-titer ACPA [18, 19]. These data suggest that the immune dysregulation that is seen in patients with RA could originate in the lungs and be related to tobacco smoke-induced citrullination of proteins, which further explain why men with RA are more likely to develop RA-ILD. Therefore, it is reasonable to hypothesize that high titers of RF and ACPA are prone to pulmonary involvement. On the other hand, interstitial lesions in RA patients and interstitial lesions of the lungs may elevate the titer of these antibodies. So, the high titer of autoantibodies may be used for early detection and as a target in the prevention from RA-ILD.

Although the specific pathogenesis of RA remains unclear, many studies have shown that the imbalance of lymphocyte subsets is an important cause of its occurrence and development. Our retrospective study found that the absolute and relative counts of CD3−CD56+ NK cells in peripheral blood of RA-ILD patients were higher than those of RA patients, while the percentage of T cells and CD4+ T cells was lower (not dramatically but significant). This suggests that the occurrence of RA-ILD may be related to the imbalance of lymphocyte subsets in patients, especially the imbalance of CD3−CD56+ NK cells and T cells.

Previous studies have shown that NK cells play a key role in the pathogenesis of autoimmune liver disease [5] and acute lung injury [6]. It can induce antifibrosis signal in the liver and lungs through two independent mechanisms: NK cells can block hepatic fibrosis by directly eliminating hepatic fibroblasts to reduce the production of collagen, and the other is by releasing antifibrotic mediators, such as interferon-*γ* (IFN-*γ*). In pulmonary fibrosis, NK cells are thought to counteract the fibrogenic activity of transforming growth factor-*β* (TGF-*β*) by producing IFN-*γ* [20]. In 2005, Esposito et al. found that the percentage and absolute number of NK cells in the peripheral blood of 11 patients with idiopathic pulmonary fibrosis (IPF) were higher than those of other interstitial pneumonia [21]. And our study did find that the number of NK cells was increased significantly in peripheral blood of patients with RA-ILD. This finding is in contrast with a previous report showing that both the percentage and absolute number of peripheral NK cells were significantly lower in IPF patients than those in healthy volunteers [22]. Result differences may be explained at least in part: Esposito et al. selected patients affected by other interstitial lung diseases and we selected patients with RA as the control group instead of healthy subjects. And the sample size may also have been an issue of concern. The above results suggest that there are two different NK environments in the course of pulmonary fibrosis in human: one in peripheral blood and another in the lungs. The expansion both in percentage and absolute number of CD3−CD56+ NK cells clearly illustrates that other mechanisms and the environment outside the lungs that may take part, or be related to RA-ILD evolution, proposes a new interesting approach to the study of that disease in humans.

T cells are considered to be important in the pathogenesis of RA [23], and some abnormalities of special T cells may be related to extra-articular disease manifestations, including lung disease [24]. Some studies also have shown that T lymphocytes may be involved in the pathogenesis of interstitial pneumonia. There may be a complex interplay among the subtypes of T cells in pulmonary fibrosis, but depending on the cell phenotype and the pulmonary microenvironment, T cells may promote or diminish the pulmonary fibrotic process [25]. For example, Sumida et al. found fibroblasts induced by Th1-mediated immune response in patients with nonspecific interstitial pneumonia [26], and Galati et al. found the imbalance of the Treg/Th17 axis in IPF patients [22]. We found that the relative count of T cells and CD4+ T lymphocytes in peripheral blood of patients with RA-ILD was lower than that of simple RA patients. It is suggested that the imbalance of lymphocyte subsets may also participate in the formation of interstitial lung in the process of the occurrence and development of primary RA, which is helpful to guide clinicians to pay attention to the multidirectional regulation of immune subsets in the treatment of RA-ILD and to restore the balance of lymphocyte subsets in order to reduce tissue damage and fibrosis.

## 5. Conclusion

In conclusion, this study confirmed that high levels of autoantibodies and imbalance of lymphocyte subsets may be involved in pro- and antifibrogenesis and the formation of interstitial lung in patients with RA, which can be used both as risk factors for the diagnosis of RA-ILD, together with older age and male. And rebalancing these T-cell subsets should alleviate inflammation and fibrogenesis in RA patients. However, the role of increase of NK cells and immunological environment outside the lungs in regulating fibrogenic and/or repair processes in RA-ILD remain to further study.

## Figures and Tables

**Table 1:** Clinical characteristics in RA-ILD and RA.

	RA-ILD (*n* = 100)	RA (*n* = 100)	*P*
Male/female	43/57	25/75	0.011
Age (year, mean ± SD)	63.89 ± 8.99	53.26 ± 13.24	0.000
Disease duration (year, mean ± SD)	9.87 ± 10.23	9.05 ± 8.28	0.941
RF (IU/ml, mean ± SD)	391.05 ± 413.34	221.02 ± 321.53	0.000
ACPA (IU/ml, mean ± SD)	828.77 ± 584.23	471.57 ± 549.60	0.000
ESR (mm/h, mean ± SD)	67.84 ± 34.42	62.88 ± 38.53	0.236
CRP (mg/L, mean ± SD)	32.72 ± 36.74	33.36 ± 37.49	0.846
IgG (g/L, mean ± SD)	14.68 ± 6.14	14.18 ± 3.85	0.953
IgA (g/L, mean ± SD)	3.32 ± 1.31	3.08 ± 1.34	0.156
IgM (g/L, mean ± SD)	1.52 ± 0.89	1.43 ± 0.77	0.424
Treatment (users/nonusers)	87/13	91/9	0.499
NSAIDs (users/nonusers)	58/42	69/31	0.142
GCs (users/nonusers)	18/82	16/84	0.851
DMARDs (users/nonusers)	11/89	22/78	0.056
GCs + DMARDs (users/nonusers)	38/62	25/75	0.067
Biological agents (users/nonusers)	4/96	11/89	0.105

ESR: erythrocyte sedimentation rate, RF: rheumatoid factor, ACPA: antibodies to cyclic citrullinated peptides, CRP: C-reactive protein, NSAIDs: nonsteroidal anti-inflammatory drugs, GCs: glucocorticoids, and DMARDs: disease-modifying antirheumatic drugs.

**Table 2:** The percentage of peripheral lymphocyte subsets in RA-ILD and RA patients.

	RA-ILD (*n* = 100)	RA (*n* = 100)	*P*
T cells (%, mean ± SD)	69.82 ± 9.30	74.45 ± 8.72	0.000
B cells (%, mean ± SD)	11.23 ± 6.27	11.65 ± 7.04	0.430
CD3−CD56+ NK cells (%, mean ± SD)	16.62 ± 8.56	12.11 ± 6.47	0.000
CD4+ T cells (%, mean ± SD)	39.44 ± 9.87	43.29 ± 9.10	0.005
CD8+ T cells (%, mean ± SD)	27.37 ± 9.64	27.37 ± 8.98	0.679
Th1 cells (%, mean ± SD)	15.71 ± 9.00	16.70 ± 12.60	0.692
Th2 cells (%, mean ± SD)	1.19 ± 0.69	1.23 ± 0.57	0.441
Th17 cells (%, mean ± SD)	0.81 ± 0.51	0.82 ± 0.47	0.568
Treg cells (%, mean ± SD)	4.66 ± 2.37	4.55 ± 2.64	0.506

**Table 3:** The absolute number of peripheral lymphocyte subsets in RA-ILD and RA patients.

	RA-ILD (*n* = 100)	RA (*n* = 100)	*P*
T cells (cells/*μ*l, mean ± SD)	1210.26 ± 533.11	1295.24 ± 543.26	0.382
B cells (cells/*μ*l, mean ± SD)	201.64 ± 163.92	205.40 ± 140.46	0.447
CD3−CD56+ NK cells (cells/*μ*l, mean ± SD)	280.40 ± 180.51	207.66 ± 148.57	0.002
CD4+ T cells (cells/*μ*l, mean ± SD)	698.54 ± 373.03	755.41 ± 310.69	0.118
CD8+ T cells (cells/*μ*l, mean ± SD)	458.50 ± 221.11	486.19 ± 284.36	0.729
Th1 cells (cells/*μ*l, mean ± SD)	107.24 ± 79.61	133.32 ± 137.98	0.652
Th2 cells (cells/*μ*l, mean ± SD)	7.99 ± 5.70	8.79 ± 5.04	0.107
Th17 cells (cells/*μ*l, mean ± SD)	5.33 ± 4.19	5.89 ± 3.93	0.136
Treg cells (cells/*μ*l, mean ± SD)	30.85 ± 22.21	32.68 ± 22.48	0.447
Th17/Treg (rate, mean ± SD)	0.22 ± 0.17	0.24 ± 0.23	0.729

**Table 4:** Multivariate analyses of factors associated with RA-ILD.

Factor	*P*	Age- and gender-adjusted	
		B-coefficient (SE)	*P*
RF	0.000	−0.001 (0.000)	0.017
ACPA	0.000	0.000 (0.000)	0.006
CD3−CD56+ NK cells (%)	0.000	−0.049 (0.023)	0.034
CD3−CD56+ NK cells (cells/*μ*l)	0.002	−0.002 (0.001)	0.04
T cells (%)	0.000	0.046 (0.019)	0.016
CD4+ T cells (%)	0.005	0.036 (0.017)	0.038

RF: rheumatoid factor and ACPA: Antibodies to cyclic citrullinated peptides.

## Data Availability

The original data used to support the findings of this study are included within the supplementary information file.

## Supplementary Materials

Supplementary Materials The supplementary material provides the original data including general information, inflammatory factors, autoantibodies, percentage, and absolute number of peripheral lymphocyte subsets of a total of 100 RA and 100 RA-ILD patients, which can support the conclusion of this study.
